# Some selenoproteins possibly involved in the development of Alzheimer’s disease: hints from the interaction proteins

**DOI:** 10.1186/1750-1326-7-S1-S32

**Published:** 2012-02-07

**Authors:** Qiong Liu, Chao Wang, Xifeng Qiao, Ping Chen, Jing Tian, Jiazuan Ni

**Affiliations:** 1 College of Life Sciences, Shenzhen University, Shenzhen 518060, P.R.China

## Background

Selenoprotein is any protein containing seleno-cysteine (Sec) encoded by UGA, a stop codon. Selenoproteins were discovered in eukaryote, bacteria and archaea, except for plants, fungi, some green alga and silk worm, because there is no selenoprotein synthesis system discovered in them. About 25 selenoproteins were found out in human being. Some selenoproteins were known as glutathione peroxidase, thioredoxin reductase, but the functions of other selenoproteins were still unknown. Some published data showed that selenium was prior enriched in brain when it is rare in food. It implied that selenium play some important roles in brain. The function of selenium was performed through selenoproteins. To investigate the biological function and molecular mechanism of selenium in human brain, yeast two hybridization was employed to screen the interaction proteins of selenoproteins in human brain library.

## Methods

Selenoprotein R (SelR), Selenoprotein K (SelK), Selenoprotein P (Selp), iodothyronine, type III (Dio3) and selenoprotein M (SelM) were cloned from human embryo kidney (HEK293) cells. The codon ‘TGA’ coding for Sec was mutated into ‘TGC’ coding for cystein (Cys). The cDNAs coding for the selenoproteins above containing Sec/Cys mutation were respectively cloned into bait vector of yeast two hybridization system. The interaction proteins of selenoproteins mentioned above were screened respectively in human fetal brain library according to the manual (Clontech, U.S.A). The interactions between selenoproteins and the prey proteins were confirmed by fluorescence/Förster resonance energy transfer (FRET) and co-immunoprecipitation (co-IP).

## Results

Two pairs of selenoprotein-protein interactions were confirmed by FRET and co-IP, including SelR and clusterin, SelM and cytochrome oxidase VI (Cox VI). Many published data showed that clusterin and CoxVI were associated with Alzheimer’s disease (AD). The interactions between Dio3 and serpin peptidase inhibitor, SelP and tubulin were confirmed by FRET and the co-IP assays were being carried out in our lab. Serpin peptidase inhibitor and tubulin were also published to be related to AD.

## Conclusion

The interactions between some selenoproteins and their interaction proteins possibly give us some hints that some selenoproteins may play some roles in the development of AD through their interaction proteins.

